# Infectious disease outbreaks drive political mistrust

**DOI:** 10.1073/pnas.2506093122

**Published:** 2025-07-17

**Authors:** Ore Koren, Nils B. Weidmann

**Affiliations:** ^a^Department of Political Science, Tobias Center for International Development, Ostrom Workshop in Political Theory and Analysis, Indiana University Bloomington, Bloomington, IN 47405; ^b^Department of Politics and Public Administration, University of Konstanz, Konstanz 78457, Germany

**Keywords:** infectious disease, political trust, epidemics

## Abstract

The COVID-19 pandemic has renewed attention to the far-reaching social implications of emerging infectious diseases, an issue with historical parallels in the transformative effects of the Black Plague and Spanish Flu. However, the potential for epidemics to reshape political trust and fuel instability has remained underexplored. This study leverages a novel dataset on zoonotic disease outbreaks—including Ebola, Marburg, H1N1, and the Black Plague—and geolocated Afrobarometer survey data from dozens of African states to investigate whether exposure to these deadly outbreaks alters public confidence in political institutions. Estimating the average-treatment-effect-in-the-treated with coarsened exact matching, we find that individuals with an infectious disease outbreak experience significant declines in trust toward the political establishment, especially the president, parliament, and ruling party (reductions of 0.2, 0.18, and 0.22 points, respectively, on a four-point scale). These findings, consistent across various spatial and temporal windows, provide robust empirical evidence that deadly infectious disease outbreaks can exacerbate political polarization and undermine political stability. The study emphasizes the critical need for policy strategies that integrate public health preparedness with efforts to preserve and rebuild institutional trust during outbreaks.

The COVID-19 pandemic reintroduced humanity to the wide social implications of emerging disease. The Black Plague has retransformed Early Modern Europe, opening the door for swift political and economic developments (1). Similarly, the Spanish Flu pandemic of 1918 was a major shaper of the new world order that emerged from the ashes of World War I (2). Yet, even in an era of growing concern about the socioeconomic and political impacts of emerging diseases, scholars paid relatively little attention to these potential relationships, especially how epidemics may shape the risk of political mistrust and instability. This is despite the fact that the study of infectious disease offers research on political outcomes a vital yet underutilized lens for advancing core theories on state capacity, trust, social policy, and exclusionary dynamics (3).

A new dataset on zoonotic disease outbreaks (4) allows us to examine whether these fears have empirical merit. “Zoonotic” refers to diseases that originate in animal hosts and spread to humans, either directly or through intermediaries, often triggering outbreaks with the potential to escalate into deadly regional or global epidemics, demanding urgent response measures. Some examples include not only the Black Plague (rodents) but also N1H1 flu (“swine flu”), Ebola (bats or primates), and Nipa (bats), among others. Considering their lethality and spread, outbreaks involving such pathogens can result in fast-spreading epidemics with pandemic risk.

## Results

To evaluate the political impact of these outbreaks, we combined outbreak data from the Geolocated Zoonotic Disease Outbreak Dataset with geolocated information from the Afrobarometer Version 7, a comprehensive database that records the political and social attitudes of individuals across dozens of African states between September 2016 and December 2018 (5). From Afrobarometer, we used questions measuring (where applicable) trust in various political actors, including the president, parliament, ruling party, and electoral commission, as well as the opposition party, police, and military.

In our geolocated sample, we identified individuals exposed to outbreaks of deadly zoonotic pathogens—including Ebola, Marburg, Anthrax, Bubonic, and Septicemic plagues, Crimean-Congo hemorrhagic fever, H1N1, H5N1, and Lassa—residing within 100 km of an outbreak’s reported location within 5 y before being surveyed. Using Coarsened Exact Matching, we matched these individuals with unaffected individuals from the same country based on socioeconomic characteristics. This produced a well-balanced sample across urban–rural locations, infrastructure (electricity, sewage, paved roads), and proximity to health centers across 4 to 28 outbreak-affected states (see *SI Appendix* file discussion). We varied the spatial (50 km, 100 km, 200 km) and temporal (2, 5, 10 y) windows, with the main analysis using the 100 km/5-y window.

**Fig. 1.**
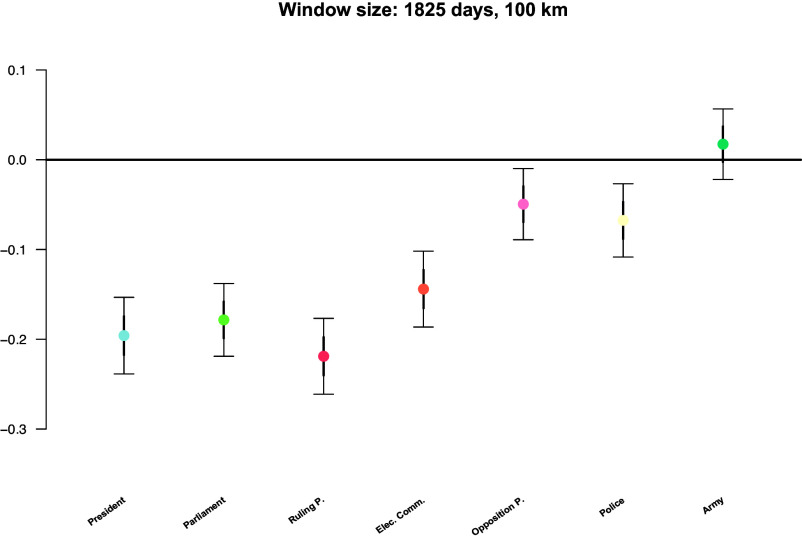
Average treatment effect in treated Afrobarometer respondents (ATT) of exposure to a zoonotic disease outbreak on trust in seven political actors/institutions. Exposure is measured by the occurrence of a disease outbreak in the respondent’s country within a radius of 100 km from the respondent’s location and in the previous 5 y from the day the survey was taken. Estimates obtained on matched samples between 10,062 and 10,454 respondents from 10 countries. Bold bars represent mean ±1 SE and whiskers represent 95% CI (two-tailed tests). Z- and *P*-values are −9.05/<0.001 (president), −8.67/<0.001 (parliament), −10.2/<0.001 (ruling party), −6.71/<0.001 (electoral comm.), −2.46/0.014 (opposition party), −3.26/0.0012 (police), and 0.870/0.384 (army).

We estimated the average-treatment-effect-in-the-treated (ATT) to assess how outbreak exposure affects political trust. Fig. 1 shows that exposure leads to significant political mistrust. Trust in the president (−0.2), parliament (−0.18), ruling party (−0.22), electoral commission (−0.14), and police (−0.07) decreases significantly (*P* < 0.01), as well as trust in the opposition party (−0.05) (*P* < 0.05). Trust in the military (0.02), an impartial bureaucracy, increases slightly but is not significant. Sensitivity tests confirm the robustness of these results with respect to trust in the president, parliament, and ruling party, across different spatial and temporal windows (n = 3,407 to 24,354, between 4 and 28 countries included, see discussion in *SI Appendix*). Including treatment–covariate interactions to further account for imbalance risk and distinguishing Ebola/Marburg from other outbreaks does not alter findings (*SI Appendix*). Since treatment and control groups could still differ in unobserved characteristics, we repeat our main analysis using a control group where only those individuals that have not been treated, but will be treated in the future, are retained. We find that the significant negative effects on trust in the president, the parliament, and the ruling party still hold (*SI Appendix*).

Additionally, a placebo test using outbreaks in neighboring countries (*SI Appendix*) shows no effect on political trust. This demonstrates that the effect does not travel across borders, and citizens’ trust in the domestic political system seems to be affected only by domestic outbreaks. In another test, we define a placebo treatment to include subjects that have not been treated in the past but will be exposed in the future. The results show that as expected, these individuals do not have lower levels of trust; if anything, they trust the government more than the ones in the control group (*SI Appendix*). This confirms again our finding that political views likely shifted negatively postoutbreak, not before.

## Discussion

Our results suggest that the effects of zoonotic disease outbreaks extend beyond mere health impacts. In postoutbreak contexts, individuals may view the government negatively for several reasons, including its failure to protect civilians from exposure and its mismanagement of the disease, its heavy-handed enforcement of containment and immobility policies, and restrictions on social practices like traditional burial rituals (3). We provide evidence consistent with a causal interpretation that such crises provoke not only public health crises but also political polarization, the erosion of trust in governance, and potential democratic backsliding, concerns that have become increasingly relevant in the wake of the COVID-19 pandemic (6). Exposure to outbreaks leads to a noticeable change in political allegiances as trust in the ruling establishment and the internal security apparatus declines. The findings align with research on the nuanced impacts of disease outbreaks on more extreme phenomena such as armed conflict (4, 7), but critically, our design underscores the broader impacts to political trust and stability during a health crisis, potentially creating fertile ground for political mobilization (8). Future research can address when and how these shifts in trust may lead to political realignments, more efficient policy responses, or even the rise of authoritarian tendencies.

For policymakers, these insights highlight the urgent need to integrate public health strategies with measures designed to preserve and rebuild political trust and point to several actionable recommendations. First, governments should integrate trust-preservation strategies into their epidemic response plans by ensuring transparency in decision-making and clear, consistent communication with the public to preempt misinformation and polarization (9). Second, crisis management should include rapid engagement with community leaders and trusted intermediaries to reinforce the legitimacy of public institutions, especially during outbreaks. Third, policymakers should invest in robust public health infrastructure and emergency preparedness that explicitly addresses the social dimensions of epidemics, including the potential for political backlash and democratic erosion. Finally, ongoing monitoring of public sentiment during health crises can help tailor responses that not only mitigate the spread of disease but also rebuild trust in political leadership and institutions, ultimately safeguarding both public health and democratic norms.

## Materials and Methods

The dependent variables measured trust in the president (question Q43a from Afrobarometer V7), parliament (question Q43b), ruling party (question Q43e), electoral commission (question Q43c), opposition party (question Q43f), the police (Q43g), and the army (question Q43h). Respondents were coded as “exposed” if an outbreak involving the following pathogens occurred within 100 km in the 5 y prior to the survey: Ebola, Marburg, Anthrax, Bubonic and Septicemic plagues, Crimean-Congo hemorrhagic fever, N1H1 and other types of swine flu, H5N1, and Lassa. Observations were matched using CEM based on whether 1) the respondent resided in a rural or an urban area (dichotomized using question URBRUR); 2) there was electric grid in the vicinity (question EA_SVC_A); 3) there were water pipes in the area (question EA_SVC_B); 4) whether there was a health clinic in the area (question EA_FAC_D); 5) the road surface at the last 5 km before the survey location was earth road or some pavement (dichotomized using question EA_ROAD_B); and 6) using binary country indicators to ensure only individuals in the same country are matched. OLS regression was used to estimate the model from which average treatment effects in the treated (ATT) were calculated in the matched sample. Our robustness checks included varying spatial/temporal windows (50/100/200 km, 2/5/10 y), pathogen type, placebo tests, and adjusting the control group.

## Supplementary Material

Appendix 01 (PDF)

## Data Availability

A detailed discussion of *Materials and Methods* appears in *SI Appendix*. Analyses were conducted in R. Additional results, replication data, and analysis scripts are available as supporting information in an OSF repository at https://osf.io/fp5y4/files/osfstorage (10).
