# Supplementary material for: Infectious disease outbreaks drive political mistrust
Source: Proc Natl Acad Sci U S A. 2025 Jul 17;122(29):e2506093122. doi: 10.1073/pnas.2506093122 (PMC12304936; doi:10.1073/pnas.2506093122)
Supplement: Supplementary file 1 — Appendix 01 (PDF) [file pnas.2506093122.sapp.pdf]

Supplementary Information for

# Infectious Disease Outbreaks Drive Political Mistrust

## Materials and Methods

**Sample** The sample was constructed from the Afrobarometer version 7 database, which surveyed 45,823 individuals in 34 African countries between September 2016 and December 2018, the periods corresponding to the temporal coverage of the Geolocated Zoonotic Disease Outbreak Database (GZOD). Because including individuals from countries that never experienced an outbreak involving one of the pathogens discussed below can bias balance statistics in the sample by inflating it with zero cases (thereby suggesting even an imbalanced sample is artificially balanced), we removed such countries from analysis before every matching run. This retained individuals from between 4 and 28 countries, depending on the geospatial and temporal window employed, that experienced at least one outbreak involving the pathogens of interest. After matching (see below), we retained a sample size of 3,407 to 24,354 respondents, depending—again—on the size of the geospatial and temporal windows and the availability of information on each dependent variable. To identify geospatial proximity to an outbreak, we received approval to use the geolocated version of the Afrobarometer database, which records coordinates of the survey's location.

**Dependent variables (DVs)** The DVs were obtained from Afrobarometer version 7 [1], and measured trust in the president (question Q43a), parliament (question Q43b), ruling party (question Q43e), electoral commission (question Q43c), opposition party (question Q43f), the police (Q43g), and the army (question Q43h).

**Exposure to zoonotic disease outbreak events** To identify our outbreak exposure treatment, we used the Geolocated Zoonotic Disease Outbreak Database (GZOD) [2]. We retained information only on those outbreaks involving the deadliest pathogens (10%-90% mortality rates), which we believe constitute the most acute threat and are most likely to involve strong state response and induce opposition. These pathogens are: Ebola, Marburg, Anthrax, Bubonic and Septicemic plagues, Crimean-Congo hemorrhagic fever, N1H1 and other types of swine flu, H5N1, and Lassa. GZOD geolocates the data based on the highest specificity available. Most observations are hence geolocated to the exact location, village or town level, and at the very least, the district level, using coordinates denoting the exact location or the district centroid. We then coded our treatment based on whether a respondent in the Afrobarometer version 7 dataset was located within a 50 km, 100 km, or 200 km radius—these geospatial boundaries are sizable enough to capture exposure even in cases where exact location was not available beyond the district level—of at least one outbreak involving these pathogens that occurred in the same country in the last two, five, and 10 years (=1), or not (=0). For simplicity, all our sensitivity tests relied on our median treatment definition, using the five year, 100 km radius.

**Matching and control covariates** To create a statistically balanced sample for estimating the ATT, we used socioeconomic indicators from Afrobarometer as matching covariates. These included: (1) whether the respondent resided in a rural or an urban area (dichotomized using question URBRUR); (2) whether there was electric grid in the vicinity (question EA_SVC_A); (3) whether there were water pipes in the area (question EA_SVC_B); (4) whether there was a health clinic in the area (question EA_FAC_D); (5) road surface at the last 5 km before the survey location (question EA_ROAD_B, dichotomized based on earth road or some pavement); and (6) binary country indicators to ensure only individuals in the same country are matched. As a precaution—as mentioned above—we removed untreated countries prior to conducting matching (excluding in the placebo test, as discussed below). These variables were also used as controls in the ATT estimation.

**Methodology** The average-treatment-effects-in-the-treated (ATT) in Figure 1 were estimated on the basis of an epidemiological causal estimation approach [2,3] in three steps. In the first step, we matched the treated units—namely, those respondents affected by an outbreak as defined above (an outbreak occurring within 50 km, 100 km, or 200 km in the last two, five, or ten years)—with untreated individuals in the same

country who share similarities across the socioeconomic and political dimensions discussed above using coarsened exact score matching (CEM). Note that we chose to use CEM rather than the "nearest" propensity score-based approach because CEM achieves balance between treated and control groups by design, eliminating the need for post-matching balance checking and reducing model dependence [4]. As illustrated in the balance plots, our matched sample is well within the balance confidence area across all indicators (absolute mean differences of <.1), suggesting the CEM functions effectively (see Additional Results, Figure S1). Additionally, CEM is computationally efficient and transparent, minimizing arbitrary matching decisions and making the process more robust than other matching methods, such as the "exact" method.

Having created a matched sample using CEM, in the second step we regressed the binary zoonotic disease outbreak treatment on each respective DV using OLS in this matched sample. CEM eliminates the need to include treatment-covariate interactions for each of the features of interest to account for the possibility that the sample was not fully balanced, although we illustrate our robustness to this decision by estimating a model where such interactions were included (see Sensitivity Tests). In the third step, we estimated and plotted ATT in the treated sample, specifically, as the change in the level of trust (on a 0 to 3 scale) for a case where outbreak exposure = 1. The reliance on matching-based methods provides estimates that are consistent with a causal interpretation in observational data such as the ones used here. All analyses were conducted in R.

**Varying Time Window Tests** To test the robustness of our findings, we first varied the spatial and temporal windows used to define outbreak exposure. Specifically, we re-estimated our models using geospatial windows of 50 km, 100 km, and 200 km from the outbreak's reported location and temporal windows of two, five, and ten years before the survey. The number of observations in these models ranged from 3,407 to 24,354. Across all these specifications, the estimated average-treatment-effect-in-the-treated (ATT) remained negative and statistically significant at the $p < .01$ level for trust in the president, parliament, and ruling party (see Additional Results, Figure S2). These results confirm that the effect of outbreak exposure on political mistrust is robust across reasonable variations in measurement.

**Sensitivity Tests** Next, we tested whether adding treatment-covariate interactions influenced the results. While interactions can account for residual imbalances in covariates, our balance plots confirmed that the matched sample already met high standards of balance. Consistent with this, including treatment-covariate interactions had little to no effect on the estimated relationships, reinforcing the robustness of our findings (see Additional Results, Figure S3). Our final set of sensitivity analyses determined whether the strength of the effect varied by pathogen type. We separated outbreaks of Ebola and Marburg, the deadliest pathogens in our sample, from all other outbreaks. As expected, the negative effect of outbreak exposure on political trust remains, substantively and statistically, practically unchanged across both disease categories (see Additional Results, Figure S4). This indicates the effects are relatively uniform across deadly pathogens. Next, since there could still be underlying differences between treatment and control group that we do not observe and that could confound the relationship we find (as for example, government neglect), we conducted an additional test where we adjust the control to include only those individuals in regions that have not been affected by outbreaks, but will be affected in the future. This design (n = 2,366-2,463, 10 countries analyzed) allows for a more credible counterfactual by limiting comparisons to units with similarly high baseline risk and potential government neglect. The results remain consistent with those in Figure 1: respondents report significantly more negative views of the president (−0.25), parliament (−0.20), and ruling party (−0.35), with all effects significant at the $p < .01$ (see Additional Results, Figure S5). Taken together, these sensitivity analyses confirm that our results are stable across different model specifications, geospatial and temporal windows, as well as pathogen severity levels.

**Placebo Tests** To further assess the validity of our findings, we conducted two placebo tests to ensure that the observed effects were not driven by unaccounted-for factors or spillover effects from neighboring countries. In the first of these tests, individuals were considered "treated" if they were exposed to an outbreak within 100 km and five years but only in a neighboring country, not their own. These individuals were then matched with untreated individuals from their own country, excluding those exposed to a domestic outbreak. Since political trust is shaped by national institutions, we expected no systematic differences in trust levels between the placebo group and their untreated counterparts. As anticipated, the

results showed no substantive or statistical effects of proximity to a foreign outbreak on any political outcomes, confirming that the main results were driven by direct exposure within a politically relevant context rather than geographic proximity alone (see Additional Results, Figure S6). The second placebo test defines a placebo treatment where individuals are "treated" if they are exposed to an outbreak in the future, but have not been exposed in the past (n = 5,798-6,196, seven countries analyzed). If the reduction in trust we find in our main analysis is due to exposure, this placebo treatment should not reduce trust. This placebo test confirms this: Consistent with expectations, no significant differences are observed in their evaluations of parliament (see Additional Results, Figure S7). However, assessments of the president and ruling party are significantly more favorable ($p < .01$ for both), while evaluations of the opposition are substantially more negative—declining by approximately 0.2 points—relative to the baseline. These patterns suggest that, pre-outbreak, individuals held, if anything, more positive pro-regime and anti-opposition sentiments on average. This further strengthens the internal validity of the findings presented in the main study.

All supplementary figures and results as well as the replication data and code are available in an OSF online repository at https://osf.io/fp5y4/files/osfstorage [5].

## References


1. Afrobarometer, Afrobarometer Round 7: Survey Data (2021). Available at https://www.afrobarometer.org.
2. O. Koren, K. N. Bukari, (Re)emerging disease and conflict risk in Africa, 1997–2019. *Nat. Hum. Behav.* **8**, 1506–1513 (2024).
3. N. Greifer, E. A. Stuart, Choosing the estimand when matching or weighting in observational studies. Preprint at https://arxiv.org/abs/2106.10577 (2021).
4. S. M. Iacus, G. King, G. Porro, Causal inference without balance checking: Coarsened exact matching. *Polit. Anal.* **20**, 1–24 (2012).
5. Weidmann NB (2025) Infectious Disease Outbreaks Drive Political Mistrust [online supplemental material]. Open Science Framework. Available at: https://osf.io/fp5y4/files/osfstorage [Last updated June 23, 2025].
